# Efficacy of artesunate-amodiaquine for treatment of uncomplicated *Plasmodium falciparum* malaria in mainland Tanzania

**DOI:** 10.1186/s12936-024-04923-0

**Published:** 2024-03-29

**Authors:** Billy Ngasala, Samwel Bushukatale, Mercy Chiduo, Twilumba Makene, Lilian Mkony, Ally Mohamed, Fablizio Molteni, Frank Chacky, Ritha J. A. Njau, Richard Mwaiswelo

**Affiliations:** 1 Department of Medical Parasitology and Entomology, School of Public Health and Social Sciences, Muhimbili University of Health and Allied Sciences, P.O. Box 65011, Dar es Salaam, Tanzania; 2 National Institute for Medical Research, Tanga Research Centre, P.O Box 5004, Tanga, Tanzania; 3 National Malaria Control Program (NMCP), Ministry of Health, P.O. Box 743, Dar Es Salaam, Tanzania; 4 Department of Microbiology, Immunology, and Parasitology, Faculty of Medicine, Hubert Kairuki Memorial University, P.O Box 65300, Dar es Salaam, Tanzania

## Abstract

**Background:**

Diversification of artemisinin-based combination therapy (ACT) is suggested as one of the strategies that can be used to contain artemisinin resistance. Artesunate-amodiaquine (ASAQ) is one of the artemisinin-based combinations that can be used in the diversification strategy as an alternative first-line treatment for uncomplicated malaria in mainland Tanzania. There is however limited data on the efficacy of ASAQ in mainland Tanzania. This study assessed the efficacy of ASAQ for treatment of uncomplicated *Plasmodium falciparum* malaria in selected sentinel sites for therapeutic efficacy studies in mainland Tanzania.

**Methods:**

Between December 2018 and March 2020, children aged between 6 months and 10 years, attending at Nagaga, Mkuzi, and Mlimba primary health facilities, and with suspected uncomplicated malaria infection were screened for eligibility to participate in the study. Malaria infection was screened using microscopy. Children with uncomplicated *P. falciparum* monoinfection and who fulfilled all other inclusion criteria, and had none of the exclusion criteria, according to the World Health Organization (WHO) guidelines, were treated with ASAQ. Follow-up visits were scheduled on days 0, 1, 2, 3, 7, 14, 21, and 28 or on any day of recurrent infection for clinical and laboratory assessment. Polymerase chain reaction (PCR)-corrected cure rate on day 28 was the primary outcome.

**Results:**

A total of 264 children, 88 in each of the three study sites (Mlimba, Mkuzi and Nagaga health facilities) were enrolled and treated with ASAQ. The ASAQ PCR-corrected cure rate was 100% at all the three study sites. None of the participants had early treatment failure or late clinical failure. Furthermore, none of the participants had a serious adverse event.

**Conclusion:**

ASAQ was highly efficacious for the treatment of uncomplicated *P. falciparum* malaria in mainland Tanzania, therefore, it can be deployed as an alternative first-line treatment for uncomplicated malaria as part of diversification strategy to contain the spread of partial artemisinin resistance in the country.

**Supplementary Information:**

The online version contains supplementary material available at 10.1186/s12936-024-04923-0.

## Background

Artemisinin-based combination therapy (ACT) remains a treatment of choice for uncomplicated *Plasmodium falciparum* malaria globally [1, 2]. Six artemisinin-based combinations have been pre-qualified by the World Health Organization (WHO) for treatment of uncomplicated *P. falciparum* malaria [2–4]. Artemether-lumefantrine (AL) and artesunate-amodiaquine (ASAQ) are the most widely adopted combinations used for ACT in sub-Saharan Africa [5]. At its introduction in early 2000s, and few years later the ACT proved to be highly efficacious [6–8]. Partial artemisinin resistance was however reported for the first time in Cambodia [9, 10], and since then it has spread to a large part of South-East Asia [11–14], and recently it has also been reported in East Africa [15–18]. Partial artemisinin resistance is conferred by point mutations in the *P. falciparum kelch*-*propeller* gene at chromosome 13 (*Pfk13*) [19]. The partial resistance is characterized by prolonged clearance of the asexual parasites after treatment-initiation, leaving a large proportion of residual parasites to be cleared by the partner-drug alone [9–11, 14, 19]. This increases the risk of selection against the long-acting partner drug, that in turn increases the risk of treatment failure [14, 20]. Anti-malarial drug resistance is also associated with increased gametocyte carriage, spread of drug resistance, morbidity, and mortality [14, 21, 22]. Resistance to chloroquine and to sulfadoxine-pyrimethamine was associated with increased morbidity and mortality, especially in children under five years of age and pregnant women in Africa [22, 23], therefore, if resistance to artemisinin is not contained it may have catastrophic effects. Anti-malarial drug resistance also has a negative effect on the economy of the malaria-endemic countries [24, 25].

In Tanzania, AL is the first-line treatment for uncomplicated malaria since December 2006 [26]. Previous therapeutic efficacy studies indicated AL to be highly efficacious and safe for treatment of uncomplicated *P. falciparum* malaria in all the malaria-endemic settings of Tanzania [27–34]. However, recently point mutations associated with partial artemisinin resistance were detected in Kagera, Manyara, Tabora, Njombe, and Coast Regions [35, 36]. It is not clear if the resistance has spread to other parts of the country. On the other hand, the cure rate following treatment with AL has fallen to 90% in Bagamoyo District, Coast Region, but without the selection of the point mutations responsible for partial artemisinin resistance [37]. No new classes of anti-malarial drugs are readily available to replace AL in ACT in case they fail. Thus, some of the available immediate short-term measures to mitigate the spread of artemisinin and partner drugs resistance include changing the first-line artemisinin-based combination or diversifying it with other available efficacious combinations in the market [2, 5, 38]. The World Health Organization (WHO) recommends that if the efficacy of the current first-line falls below 90% it should be replaced by another artemisinin-based combination with an efficacy of above 95% [2, 5, 38]. The WHO also recommends diversification of ACT, and this involves concurrent deployment of multiple artemisinin-based combinations in a malaria-endemic country [2, 5]. ASAQ is a potential combination that can replace or be diversified with AL in Tanzania. Some previous studies in mainland Tanzania, Zanzibar and other parts of Africa have indicated ASAQ to be more efficacious and well-tolerated than AL, while other studies have indicated AL to be more efficacious, and others have indicated that the two drugs have similar efficacy [27, 39–44]. Furthermore, the two drugs have antagonistic resistance mechanisms, whereby ASAQ selects for the *P. falciparum multidrug resistance* gene (*Pfmdr1*) 1 86Y, Y184, and 1246Y, and the *P. falciparum chloroquine transporter* gene 76 T (*Pfcrt)*, while AL selects for *Pfmdr1* N86, 184F, and 1246D and *Pfcrt* K76 [45–47]. Following years of widescale use of AL in Tanzania there is a significant increase in the prevalence of *Pfmdr1* N86, 184F, and 1246D and that of *Pfcrt* K76 [46], thus the parasites are expected to be more sensitive to ASAQ. Moreover, unlike AL, ASAQ is a three-doses, once a day for three days, hence this may improve the compliance. This study, therefore, assessed the efficacy of ASAQ in three sentinel sites in Tanzania for treatment of uncomplicated *P. falciparum* malaria.

## Methods

### Study area

**Fig. 1:**
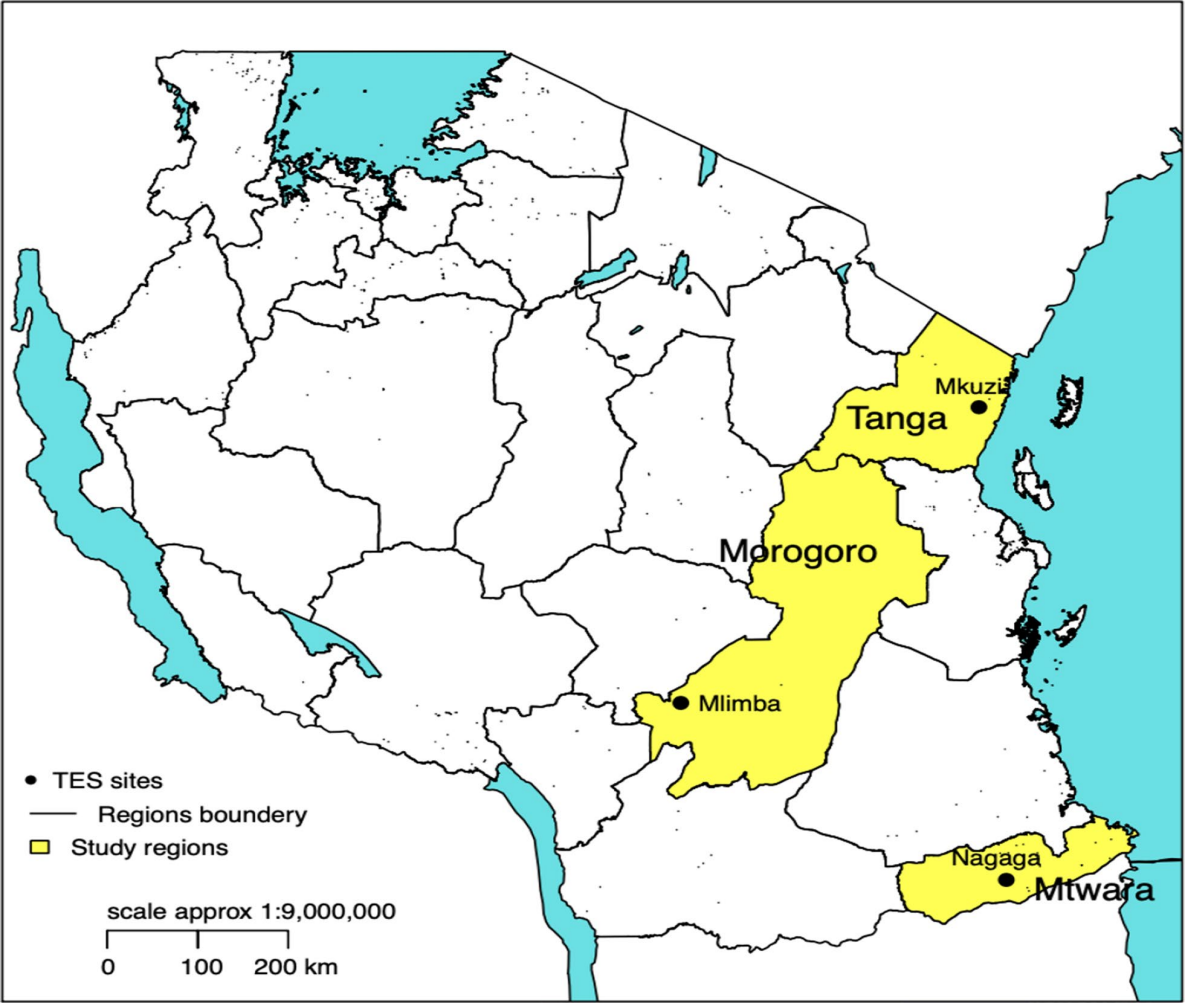
The map of Tanzania showing the study sites

The study was conducted between December 2018 and March 2020 at Nagaga, Mkuzi, and Mlimba primary health facilities in Masasi, Muheza, and Kilombero Districts, respectively (Fig. 1). The health facilities are among the sentinel sites of the National Malaria Control Programme. The major economic activities in these study sites include subsistence farming, petty trade, fishing, and small-scale mining.

The Districts have favourable conditions for *Anopheles* mosquitoes to thrive and transmit malaria. Masasi District in Mtwara Region has an annual rainfall averaging 939 mm and temperature of 25.4 °C. The rainy season is between January and April. Muheza District is in Tanga Region, and it has the average annual rainfall of 101.9 mm and temperature of 28.6 °C. On the other hand, Kilombero District in Morogoro Region has the average rainfall of 1350 mm and temperature of 26 °C. *Plasmodium falciparum* is the predominant malaria parasite, and *Anopheles arabiensis* the major vector in the study sites. Malaria transmission in the study areas occurs throughout the year with peaks corresponding to the long and short rainy seasons. The major malaria control measures in the Districts include insecticide-treated bed nets, diagnosis and treatment with ACT, and intermittent preventive treatment in pregnant women using sulfadoxine-pyrimethamine.

## Study design

This was a single-arm prospective study for assessing the therapeutic efficacy of ASAQ as part of routine therapeutic efficacy studies (TES) conducted after every two years in sentinel sites to assess the efficacy of the first-line and alternative first-line treatments for uncomplicated malaria in the country. Patients presenting at the health facilities with suspected acute uncomplicated malaria were screened for eligibility. Eligible patients with microscopy-confirmed uncomplicated *P. falciparum* mono-infection were enrolled in the study, treated with artesunate-amodiaquine, and thereafter, followed up until day 28 after treatment-initiation for the assessment of clinical and parasitological responses. The therapeutic failures were classified as early treatment failure (ETF), late clinical failure (LCF), and late parasitological failure (LPF) [48]. ETF was defined as presence of danger signs or severe malaria on day 1, 2 or 3, in the presence of parasitaemia; or parasitaemia on day 2 higher than on day 0, irrespective of axillary temperature; or parasitaemia on day 3 with axillary temperature ≥ 37.5 °C; or parasitaemia on day 3 ≥ 25% of count on day 0. LCF was defined as presence of danger signs or severe malaria in the presence of parasitaemia on any day between day 4 and day 28 (day 42) in patients who did not previously meet any of the criteria of early treatment failure; and presence of parasitaemia on any day between day 4 and day 28 (day 42) with axillary temperature ≥ 37.5 °C in patients who did not previously meet any of the criteria of early treatment failure. LPF was defined as presence of parasitaemia on any day between day 7 and day 28 (day 42) with axillary temperature < 37.5 °C in patients who did not previously meet any of the criteria of early treatment failure or late clinical failure. On the other hand, the adequate clinical and parasitological response (ACPR) was defined as the absence of parasitaemia on day 28 irrespective of axillary temperature, in patients who did not previously meet any of the criteria of ETF, LCF or LPF [48].

## Study population and recruitment

The patients were included in the study if had the following criteria: age from 6 months to 10 years, weight ≥ 5 kg, body temperature ≥ 37.5 °C or history of fever in the last 24 h, microscopy confirmed *P. falciparum* mono-infection, parasitemia level of 250–200 000/µL, ability to swallow oral medication, ability and willingness of the caregivers to abide by the study protocol and the stipulated follow-up visits and, a written informed consent from a caregiver and assent from children aged ≥ 7 years. Children aged above 5 years were included in the study to accelerate recruitment rate since malaria prevalence has declined significantly in many parts of the country especially among children aged below five years. Potential participants were excluded in case of the evidence of severe malaria or danger signs, known allergy to the study medicines, reported anti-malarial intake within the past two weeks, blood transfusion within last 90 days, presence of a febrile condition other than malaria, and known underlying chronic or severe disease including severe malnutrition.

At the facilities patients and their caregivers were informed in detail about the study, including its purpose and the associated risks and benefits, and their rights. Patients were evaluated clinically, and those with signs/ symptoms of malaria were tested for malaria infection. Thereafter, caregivers of the children with microscopy-confirmed uncomplicated *P. falciparum* malaria infection, and who had met all the inclusion and none of the exclusion criteria were invited to participate in the study, and were then administered with the consent form. The consent form was self-administered to the literate caregivers, and it was administered by the study clinician to the illiterates. All the procedures were performed in the presence of a witness to ensure that there is no coercion. The caregivers were given time to ask questions to their satisfaction. Consented caregivers signed the form either by a written signature for literates or a thumbprint together with a written signature of their witnesses for the illiterates.

## Treatment

The patients were treated using a standard weight-based 3-day once a day course of co-formulated artesunate-amodiaquine [Winthrop®] tablets containing 25 mg artesunate and 67.5 mg amodiaquine or 50 mg artesunate and 135 mg amodiaquine. The WHO in Tanzania donated ASAQ tablets for the study. The doses were administered based on weight-bands as follows: For ASAQ (25/67.5 mg), 1 tablet was given to children weighing < 10 kg; and for the 50/135 mg tablets, 1 tablet was given to those with 10–20 kg; 2 tablets to children with 21–30 kg, and 3 tablets to children weighing > 30 kg. The ASAQ dispersible tablets suspended in water were administered to the children who were unable to swallow the whole tablet(s). Milk containing biscuits were administered before each dose of the artesunate-amodiaquine. Food improves the absorption of amodiaquine. All the treatment doses were directly observed. A study nurse administered the medicines and observed the patients for 30 min after each dose. A full dose was readministered in case of vomiting within this period. The patients with fever over 38˚C were treated with paracetamol. The caregivers were also instructed to use tepid sponging to lower body temperature in children aged below five years.

## Patient withdrawal

A participant would be withdrawn from the study in case of vomiting the study drug > 3 times, withdrawal of consent, the onset of a serious febrile illness, intake of any drug with anti-malarial properties outside the study protocol, or any protocol violation. In case a participant missed the scheduled follow-up visit and did not show up on the successive days despite the tracing efforts, was considered lost to follow-up and was consequently censored from the analysis on the last day he/she was seen. The participant who returned before the last day of follow-up was not considered as lost to follow-up. The patients with symptoms/signs of severe disease (including repetitive vomiting of study drug) were managed in accordance with the Tanzania National Guidelines for Treatment of Malaria and followed up until recovery, but were censored from the analysis on the day of withdrawal [49]. Patients with non-severe clinical or parasitological failure after day 14 were treated with artemether-lumefantrine.

## Procedures

The assessment for clinical and laboratory parameters were performed on days 0, 1, 2, 3, 7, 14, 21, and 28 or any day of recurrent illness. All the clinical and laboratory data were recorded in a case record form.

### *Clinical and safety assessments*

It involved taking a history of clinical symptoms and signs, possible adverse events (AEs), concomitant drug consumption, and clinical examination including measurement of axillary temperature for fever. Fever was defined as a body temperature ≥ 37.5 °C.

Safety assessment involved direct questioning of the caregivers and recording the nature and incidence of AE and/or serious adverse events (SAE) that might have occurred after taking the study medicine. The caregivers also received an information card with instructions on how to identify signs and symptoms of commonly reported AEs. Signs and symptoms such as fatigue, weakness, dizziness, headache, palpitations or allergic drug reactions (rash), diarrhoea, abdominal pain, dizziness, itching, and fever were recorded before, during, and after treatment. The clinical signs and symptoms recorded during and after treatment were compared with those recorded before treatment to determine if they are related to the study medicine. Patients with AE were kept under observation, and those requiring treatments were treated accordingly.

An AE was defined as any unfavourable sign, symptom, syndrome, or disease that developed or worsened during the study even if it was not considered to be related to the study drug, whereas a SAE was defined as any untoward medical occurrence that was life-threatening; required hospitalization or prolongation of hospitalization; resulted in a persistent or significant disability or incapacity or resulted in death [50]. The AEs were further classified into mild, moderate, severe, and life-threatening as follows: mild—easily tolerated, no or minimal interference with daily activities; moderate-low level of inconvenience, greater than minimal interference with daily activities; severe—interrupts normal daily activities, usually incapacitating; life-threatening – life-threatening consequences, urgent intervention indicated, or death.

### Laboratory assessment

#### Blood sampling

Finger-prick capillary blood samples were collected and used to prepare thick smears for microscopy to assess presence and density of asexual parasitaemia and gametocytaemia, thin-smears for microscopy to determine the species of infecting malaria parasites, and also blotted on a 3 MM Whatman filter paper for molecular analysis to differentiate recrudescence from new infection. Filter papers containing dried blood spots were labelled relevant patient and study information, air-dried at room temperature for 3–4 h, packed in individual plastic bags, and then stored in a cabinet at room temperature. At the end of the study the filter papers were transported to Muhimbili University Parasitology Laboratory, Dar es Salaam, Tanzania where they were stored waiting for molecular analysis.

#### Malaria microscopy

During the study, thin smears were prepared at the enrolment only, whereas the thick smears were prepared at all the sampling time points. Two pairs of thin and thick smears were prepared at the enrolment. The first pair was quickly stained using 10% Giemsa for 15 min to accelerate the patients’ enrolment, whereas the second pair was stained slowly using 3% Giemsa for 1 h. Likewise, the thick smears collected during follow-up were stained slowly using 3% Giemsa for 1 h. For quantification, the asexual and sexual parasites on thick smears were counted against 200 white blood cells (WBC). The obtained number was multiplied by 40 to gain an approximate parasite density per microlitre of blood, assuming that a microlitre of blood contains 8000 leukocytes. If no parasite was seen after examining 100 fields then a blood smear was considered negative. All the slides were read by two independent microscopists. A third independent reading was performed in case the microscopists disagreed on the presence of parasitaemia or if the density of the parasites differed by more than 25%. Likewise, in the case of positive versus negative results, a third independent reading was used to confirm the reading of the first two readers.

#### Molecular analysis

In order to distinguish recrudescent infection from newly acquired infection (reinfection), molecular analysis was completed on the day 0 and day of failure samples for participants who experienced clinical or parasitological failure during the 28-day follow-up period. DNA was extracted from DBS using ZymoBIOMICS DNA Mini Kit (Zymo Research, California, USA) according to manufacturer’s instructions. Paired samples were genotyped by analyzing the merozoite surface proteins (*msp*) 1 and 2, and glutamine-rich protein (*glurp*) using the standard protocol. All three markers were assessed for all samples. Amplicons were run on a 2% agarose gel and images of the gels were captured.

PCR images were viewed in Microsoft Word as image quality was greatly reduced during printing. A rectangle was inserted into the document and acted as a ruler. Fragment length estimates were evaluated independently by two trained staff. Bands were considered a match if the day 0 and day of failure fragment lengths were within 10 base pairs for *msp1* and *msp2* and within 50 base pairs for *glurp*.

Reinfection and recrudescence were differentiated using both the 3/3 and 2/3 methods as recommended by the WHO. If there was at least one matching band in any sub-allele for all three makers the recurrent infection was classified as a recrudescence with the 3/3 method. If there was at least one matching band at any sub-allele for two of the three markers, the recurrent infection was classified as a recrudescence with the 2/3 method. If insufficient matches were identified, the recurrence was classified as reinfection. If there were no amplification products resulting in sharp, defined bands in both the pre-treatment and day-of-failure samples for a gene, that gene was not used to distinguish between recrudescence and reinfection.

## Study outcomes

The primary outcome was the proportion of patients with PCR-corrected ACPR by day 28. Secondary end-points included: proportion of patients who have cleared fever on days 1, 2 and 3, proportion of patients who have cleared asexual parasites on days 1, 2, and 3, proportion of patients with early treatment failure, proportion of patients with late clinical failure, proportion of patients with late parasitological failure, and proportion of patients with serious adverse events.

**Ethical approval.**

During the conduct of the study the Good Clinical Practices, the Declaration of Helsinki, and applicable regulatory requirements in Tanzania were observed. The protocol was approved by the National Research Coordinating Committee of the National Institute for Medical Research and the Institutional Ethics Review Board of the Muhimbili University of Health and Allied Sciences. The permission to conduct the study at Nagaga, Mkuzi, and Mlimba health facilities was obtained from the respective authorities in Districts and Villages in which the primary health facilities are located. A written informed consent was obtained from the caregivers of the participants whereas children aged ≥ 7 years also provided assent before their enrollment.

## Sample size and data analysis

The WHO guideline was used to calculate the sample size [48]. Briefly, with the assumptions of an expected proportion of treatment failures in the study population of 5%, a confidence level of 95%, and a precision level of 5%, thus, a minimum of 73 patients were to be enrolled. An attrition rate of 20% was added to account for patients who were likely to be either lost during follow-up, withdraw or would be excluded after detection of reinfection with PCR correction. Thus, 88 patients were enrolled in each study site.

The data were double-entered in an electronic database, cleaned, and analysed using the Statistical Package for Social Sciences (SPSS) software version 22 (SPSS Inc, Chicago, USA) as per protocol. The means were compared using independent sample *t*-test. *Chi* square or Fisher’s tests were used to compare proportions. The cure rate end point was analysed using survival analysis. The data were censored at the time of withdrawal for patients lost to follow up, withdrawal of consent and PCR determined re-infection or uncertain PCR outcome. A *p* ≤ 0.05 was considered significant.

## Results

### Baseline characteristics of the study participants

**Fig. 2:**
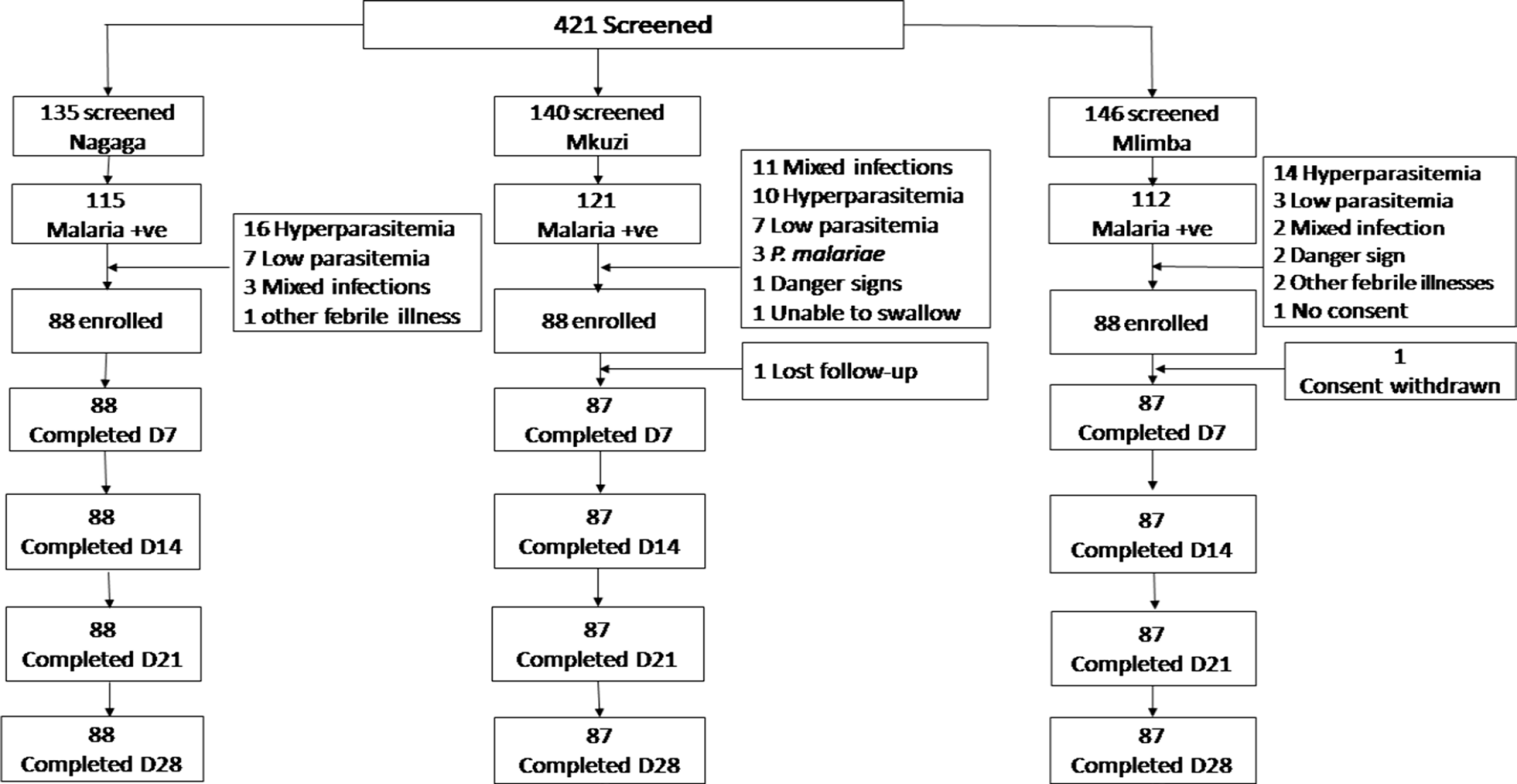
Study profile

A total of 421 children aged between 6 months and 10 years were screened for eligibility to participate in the study. Two hundred and sixty four (62.7%) eligible children were enrolled in the study. The study profile is presented in Fig. 2. The baseline characteristics of the study participants are presented in Table 1. Participants in all the study sites had similar baseline characteristics except the mean axillary body temperature, and mean parasite density.

**Table 1:** Baseline characteristics of the study participants

Characteristics	Overall	Nagaga	Mkuzi	Mlimba	P-value
	n = 264	n = 88	n = 88	n = 88	
Females, n (%)	144 (54.6)	48 (54.6)	49 (55.7)	47 (53.4)	0.955
Age (years), Median (IQR)	4 (2–7)	4 (2.5–6)	5 (3 -7)	3 (2–7)	0.355
History of fever past 24 h	260 (98.5)	88 (100)	85 (96.6)	87 (98.9)	0.169
Temperature C, Mean (SD)	38.3 (1.5)	38.3 (1.3)	37.8 (1.8)	38.3 (1.3)	0.05
Geometric mean parasite density (/μL)	30,361 (25,862–35,642)	23, 785 (17,142–33,004	44,880 (36,320–55,458)	26,216 (19,936–34,473)	0.003
Parasite density range (/μL)	400–184,480	400–184,480	920–159,720	640–183,080	NA

*IQR* Interquartile range; ^*0*^*C* Celsius degree; *SD* Standard deviation, *μL* microlitre

## Fever and parasites clearance

At the enrolment 66.3% (175/264) of the participants were febrile. Following the initiation of ASAQ treatment, only 5.3% (14/263), 0.4% (1/264) and 0.4% (1/263) of the participants remained febrile on days 1, 2, and 3, respectively. Parasite clearance is presented in Table 2. Less than 20% and none of the patients were having parasitemia on days 2 and 3, respectively. Parasite clearance was rapid in Mlimba than in the other two sites, although it was not statistically significant.

**Table 2:** Parasite clearance by microscopy and study site

Characteristics	Overall		Nagaga		Mkuzi		Mlimba		
	n (%)	95% CI	n (%)	95% CI	n (%)	95% CI	n (%)	95% CI	
Day 0	264 (100.0)	NA	88 (100)	NA	88 (100)	NA	88 (100)	NA	
Day 1	232 (87.8)	84–92	76 (86.3)	77–92	83 (94.3)	87–97.6	73 (83)	74.5–90.3	
Day 2	36 (13.6)	10–18.3	15 (17)	10.4–26.2	12 (13.6)	7.9–22.6	9 (10.2)	5.4–18.8	
Day 3	0 (0)	NA	0 (0)	NA	0 (0)	NA	0 (0)	NA	
Geometric Mean Parasite density (/μL)									
Day 0	30,360	25,862–35,642	23, 785	17,142–33,004	44,880	36,320–55,458	26,216	19,936–34,473	
Day 1	501	388–648	742	488–1129	431	257–722	396	273–575	
Day 2	46	26–81	28	20 -39	114	22–597	33	20–56	
Day 3	NA	NA	NA		NA	NA	NA	NA	NA

*μL*, microlitre; *CI*, confidence interval, *NA*, not applicable

## Treatment outcomes

None of the patients had early treatment failure or late clinical failure following the administration of ASAQ. Two patients had late parasitological failure on day 28, one at Nagaga and the other at Mkuzi. The PCR-corrected cure rate was 100% at all the study sites by using 3/3 method for differentiation of recrudescence from new infection. The treatment outcomes are presented in Table 3. Furthermore, none of the participants had a serious adverse event.

**Table 3:** Parasitological and clinical outcome for patients treated with artesunate-amodiaquine

Outcome	Nagaga ( n = 88)	Mkuzi (n = 88)	Mlimba (n = 88)
	n (%)	n (%)	n (%)
Early treatment failure	0 (0)	0 (0)	0 (0)
Late clinical failure	0 (0)	0 (0)	0 (0)
Late parasitological failure	1 (1.1)	1 (1.2)	0 (0)
Uncorrected adequate clinical and parasitological response	87 (98.9)	86 (98.9)	87 (100)
PCR-determined reinfection by day 28	1 (1.1)	1 (1.1)	0
Recrudescence	0	0	0
Withdrawn/lost	0	1 (1.1)	1 (1.1)
PCR-corrected adequate clinical and parasitological response	88 (100)	87 (100)	87 (100)

## Discussion

Artemether-lumefantrine (AL) is the first-line treatment for uncomplicated *P. falciparum* malaria in mainland Tanzania since 2006 [26]. However, the recent reports in Tanzania on the presence of point mutations responsible for partial artemisinin resistance in Kagera, Manyara, Tabora, Njombe, and Coast Regions [35, 36], and the decline of AL cure rate in Bagamoyo District, Coast Region [37], are worrisome. Thus, a mitigation plan is needed to alleviate the increased morbidity and mortality that would ensue in case the AL fails. The aim of this study was, therefore, to assess the efficacy of ASAQ as a potential ACT that can be used in the diversification strategy with AL and other artemisinin-based combinations. The findings in this study showed that ASAQ was very efficacious with all the three study sites having a cure rate of 100%. Similar high cure rates have been reported in the previous studies conducted in mainland Tanzania [27, 40], and other malaria-endemic countries [39, 41–44, 51–58]. The WHO recommends a new anti-malarial drug to have a cure rate of 95% and above for it to be acceptable as first-line treatment for uncomplicated falciparum malaria. With the efficacy of 100% in the three sentinel sites, ASAQ qualifies to be a first-line treatment in Tanzania. In addition, none of the participants had early treatment failure or late clinical failure. Fever and parasite clearance were also very rapid. Ninety two percent of the participants who were febrile at baseline were afebrile on day 1, and only 0.4% of them remained febrile on days 2 and 3. For the parasite clearance, less than 20% of the participants had parasitemia on day 2, and none of the participants was parasitemic on day 3. Furthermore, compared to Nagaga and Mkuzi, Mlimba had the lowest proportion of participants with parasitemia on days 1 and 2, although not statistically significantly different. In addition, although not statistically significant, a large proportion of participants had parasitemia on day 1 at Mkuzi than it was at Nagaga and Mlimba. Initial parasite density is among the factors that determine parasite clearance after treatment initiation [46]. Patients in Mkuzi had significantly higher initial geometric mean parasite density than those in the other two sites. Therefore, initial parasite density may explain the higher proportion of patients with parasitemia on day 1 at Mkuzi and the lower proportion of patients with parasitaemia at Mlimba site both on day 1 and day 2 after the treatment initiation. On the other hand, ASAQ was well-tolerated and safe in all the three study sites. None of the study participants had a serious adverse event.

## Conclusion

ASAQ was highly efficacious for the treatment of uncomplicated *P. falciparum* malaria in mainland Tanzania, therefore, it can be deployed as alternative first-line treatment or as part of diversification strategy to contain the spread of partial artemisinin resistance in the country which requires the use of multiple first-line artemisinin-based combinations.

### Supplementary Information

**Additional file 1: Table S1.** Genotyping results for differentiation of recrudescence from new infection. A difference of  more than 10 base pairs in bands size for msp 1 and 2, and of more than 50 base pairs for glurp was used to differentiate recrudescence from new infection.

## Data Availability

All the relevant data are within the manuscript and its Additional file 1: Table S1.

## Abbreviations

ACPR: Adequate clinical and parasitological response
AE: Adverse events
AL: Artemether-lumefantrine
ASAQ: Artesunate-amodiaquine
CI: Confidence interval
ETF: Early treatment failure
LCF: Late clinical failure
LPF: Late parasitological failure
NMCP: National Malaria Control Programme
*Pfk13*: *Plasmodium falciparum* Kelch 13
*Pfcrt*: *Plasmodium falciparum* Chloroquine resistance transporter
*Pfmdr*: *Plasmodium falciparum* Multidrug resistance
PCR: Polymerase chain reaction
SAE: Serious adverse event
SPSS: Statistical package for social sciences
TES: Therapeutic efficacy study(s)
WBC: White blood cells
WHO: World Health Organization
