# Supplementary material for: Efficacy of artesunate-amodiaquine for treatment of uncomplicated Plasmodium falciparum malaria in mainland Tanzania
Source: Malar J. 2024 Mar 29;23:90. doi: 10.1186/s12936-024-04923-0 (PMC10979577; doi:10.1186/s12936-024-04923-0)
Supplement: Supplementary file 1 — Additional file 1: Table S1. Genotyping results for differentiation of recrudescence from new infection. A difference of more than 10 base pairs in bands size for msp 1 and 2, and of more than 50 base pairs for glurp was used to differentiate recrudescence from new infection. [file 12936_2024_4923_MOESM1_ESM.docx]

**Additional file 1**

**Table S1:** Genotyping results for differentiation of recrudescence from new infection. A difference of ≥ 20 base pairs in bands size was used to differentiate recrudescence from new infection.

| **Patient** | **Day** | **Msp1** | | | **Msp2** | | **Glurp** |
| --- | --- | --- | --- | --- | --- | --- | --- |
|  |  | KI | MAD20 | RO33 | FC27 | 3D7/IC |  |
| 1 | D0 | 209 | 252 | 168 | 564 | 536 | 875/989 |
|  | D28 | 177/240 | 0 | 0 | 334/459 | 653 | 1021 |
| 2 | D0 | 288 | 190 | 0 | 508 | 629 | 714 |
|  | D28 | 214 | 0 | 0 | 485 | 585 | 844 |
